# Involuntary Treatment in Patients with Eating Disorders

**DOI:** 10.1192/j.eurpsy.2025.701

**Published:** 2025-08-26

**Authors:** R. F. Dionísio, C. Portela, D. Areias, S. M. Sousa, C. Fonseca, C. P. Desport

**Affiliations:** 1 Hospital de Magalhães Lemos, Unidade Local de Saúde de Santo António, Porto, Portugal

## Abstract

**Introduction:**

Eating disorders, in particular Anorexia Nervosa (AN), are serious psychiatric disorders with a chronic course and high levels of disability and mortality. These disorders are characterized by a misrepresentation of body image and intense fear of putting on weight, leading to cognitive distortions related to food and weight control, as well as dysfunctional behaviors aimed at weight loss.

**Objectives:**

The aims of this paper are to provide a summary of the current literature concerning involuntary treatment in patients with eating disorders and to assess whether there is a difference in terms of baseline characteristics and treatment outcomes between patients treated both voluntarily or involuntarily.

**Methods:**

Relevant articles were identified by searching the following terms: “treatment refusal”, “involuntary/compulsory/coercive/forced treatment/admission”, “eating disorders”, “anorexia nervosa”, “bulimia nervosa”. Research was restricted to articles concerning humans and published between 2014 and 2024 in English.

**Results:**

The treatment of eating disorders consists in a combination of weight control/weight gain methods and psychotherapy, as well as the treatment of organic complications associated with starvation and low body weight.

Involuntary treatment is usually intended for patients having worse baseline conditions. Factors associated with increased involuntary treatment utilization were female sex, lower age, psychiatric comorbidities, more severe disease with lower weight at admission and a longer course.

Concerning short term outcomes, the involuntary treatment can be life saving and half of these patients accept the treatment in 2 weeks time. Studies show that the involuntary treatment did not have a significant negative impact in the doctor-patient therapeutic relation. In terms of long term outcomes, patients treated involuntarily had similar outcomes to those treated voluntarily, having better outcomes in some domains, including menstruation, number of admissions and functionality.

**Conclusions:**

The denial of the illness and lack of insight in AN raise practical and ethical questions relating to the autonomy of the patient and the responsibility of the family and health care practitioners. The involuntary treatment of eating disorders is a complex area and further research including quantitative and qualitative studies is needed.

**Disclosure of Interest:**

None Declared

